# Executive functioning and binge-watching behaviors in adolescents: a network analysis

**DOI:** 10.3389/fpubh.2026.1933368

**Published:** 2026-09-29

**Authors:** Jorge Becerra-Arroyo, Nicolás Ruiz-Robledillo, Rosario Ferrer-Cascales, Borja Costa-López, Sara Rodríguez-Espinosa, Natalia Albaladejo-Blázquez

**Affiliations:** Department of Health Psychology, Faculty of Health Sciences, University of Alicante, Alicante, Spain

**Keywords:** adolescents, binge-watching, executive functioning, gender, network analysis

## Abstract

**Background:**

Binge-watching (BW) is a prevalent form of media consumption among adolescents, yet its cognitive correlates remain insufficiently understood. This study examined the relationships between BW behaviors and executive functioning in a sample of 940 adolescents, while also exploring gender-specific patterns (536 males, 404 females; *M* = 15.44 years, SD = 0.77).

**Methods:**

Participants completed an elaborated ad hoc sociodemographic questionnaire, the Binge-Watching Engagement and Symptoms Questionnaire (BWESQ), the Teenage Executive Functioning Inventory (TEXI), and the Family Affluence Scale (FAS), which was used to assess participants’ socioeconomic proxy. Descriptive analyses revealed that females reported higher engagement, positive emotions, and desire-savoring scores, while females also reported slightly higher executive functioning difficulties. Partial correlations controlling for age, gender, and FAS showed that executive dysregulation, particularly inhibition and working memory deficits, was positively associated with problematic binge-watching dimensions. Network analyses were conducted using Gaussian Graphical Models estimated with EBICglasso, and gender differences were examined through the Network Comparison Test (NCT).

**Results:**

Network analysis highlighted the central role of affective and control-related BW dimensions, revealing interdependent relationships between cognitive difficulties and binge-watching behaviors. Although global network strength was comparable across genders, network structure differed significantly between males and females, suggesting gender-related differences in the organization of associations between executive functioning and binge-watching dimensions.

**Discussion:**

These findings underscore the multidimensional nature of adolescent binge-watching, highlighting how executive functioning deficits may act as a vulnerability factor for dysregulated consumption patterns. The results may inform future prevention strategies and emphasize the importance of considering cognitive factors when examining adolescent media use from a gender perspective.

## 1 Introduction

Binge-watching (BW), commonly defined as the consumption of multiple episodes of a television series in a single sitting (1), has become a highly prevalent media consumption behavior among adolescents (2–4). Epidemiological evidence suggests that between 30 and 60% of adolescents engage in BW regularly (5–7). In Spain, data from a nationally representative sample of more than 17,000 adolescents showed that 46.3% accessed streaming platforms daily, while 40.8% did so several times per week (8). Although such indicators do not directly measure BW episodes, they indicate a context of frequent exposure to on-demand audiovisual content among Spanish adolescents.

Previous research conceptualizes BW as a heterogeneous behavior ranging from recreational, controlled viewing to more dysregulated patterns associated with loss of control and functional impairment (1). For many adolescents, BW may represent a recreational and socially shared activity associated with entertainment, relaxation, and emotional engagement (1). However, excessive or poor viewing may interfere with daily functioning, sleep routines, academic responsibilities or emotional regulation, reflecting more maladaptive forms of engagement (1, 4, 9, 10). This distinction is particularly relevant in adolescence, a developmental period characterized by ongoing maturation of executive control systems and heightened sensitivity to reward and affective cues (11).

Within this framework, increasing attention has been directed toward the role of Executive Functioning (EF) difficulties in explaining variability in BW behaviors. EF deficits, particularly in inhibitory control and working memory, are central to self-regulation and goal-directed behavior (12). In adolescents, executive dysregulation may hinder the capacity for managing viewing behavior, such as interrupting ongoing episodes, resisting automatic playback, or maintaining long-term goals in the presence of immediate entertainment rewards (13, 14). However, empirical evidence linking specific EF difficulties to BW remains limited, and most studies have relied on global EF indices, potentially obscuring heterogeneity in the associations between distinct executive deficits and the multidimensional structure of BW behaviors (15–17). This limitation is particularly relevant because BW encompasses both adaptive engagement and dysregulated patterns, whose specific cognitive correlates remain insufficiently understood.

Sociodemographic factors may further influence BW patterns and their cognitive correlates. Previous research suggests that gender differences exist in levels of engagement, with females often reporting higher involvement, although findings remain inconsistent regarding whether these differences reflect behavioral intensity or differences in self-regulatory processes (2, 4). Socioeconomic status may also influence patterns of media consumption and was therefore considered as a covariate in the present study (18, 19). Importantly, sociodemographic variables may also contribute to variability in the relationship between cognitive control and problematic BW behaviors during adolescence (20). Given these gaps, the present study aims to (1) examine the associations between binge-watching dimensions and executive functioning difficulties, focusing on inhibitory control and working memory deficits using both correlational and network-based approaches, and (2) investigate potential gender differences in the network structure linking BW and executive functioning. By adopting a network-based perspective, this study moves beyond traditional variable-centered approaches to examining the system-level organization of relationships between cognitive and behavioral dimensions underlying adolescent BW. Unlike classical regression models, which primarily estimate the contribution of individual predictors to an outcome variable, network analysis allows the examination of the interconnected structure among multiple dimensions simultaneously, identifying central and bridging nodes that may contribute to the organization of complex behavioral patterns.

Based on previous literature, it was hypothesized that (H1) executive functioning difficulties, specifically inhibitory control and working memory deficits, would be positively associated with BW behaviors, showing stronger and more central associations with dysregulated, problematic dimensions (e.g., dependency and loss of control) than with recreational engagement. Furthermore, we hypothesized that (H2) the network architecture linking BW and executive deficits would exhibit significant structural variations between males and females, reflecting gender-specific differences in how cognitive control and affective dimensions interact during media consumption (2, 4, 9, 13, 14).

## 2 Materials and methods

### 2.1 Participants

The study employed a cross-sectional observational design to examine binge-watching behavior and executive functioning among Spanish adolescents attending public and private secondary schools.

Inclusion criteria required participants to be enrolled in the academic grade targeted by the study, being at least 12 years old, being present in the classroom on the day of the data collection and providing informed assent along with parental or legal guardian consent. Exclusion criteria included not belonging to the target academic grade, absence on the day of assessment, lack of consent, withdrawal from the study, and cases with incomplete or unclassifiable sociodemographic information.

A school-based cluster sampling method was employed, with secondary schools constituting the sampling clusters. The clusters were selected to ensure coverage of the main geographical areas represented in the target population. A total of 23 secondary schools were selected and invited to participate, and all agreed to take part in the study. Within the selected clusters, all eligible students were invited to participate. Following the applicable informed consent and assent procedures, the initial participating sample comprised 950 eligible students. Ten cases were subsequently excluded because of missing sociodemographic information, resulting in a final analytical sample of 940 adolescents (98.9% of the initial participating sample) aged 14–18 years (*M* = 15.44, SD = 0.77). The sample size was therefore determined by the cluster-based recruitment strategy and the number of eligible students available within the selected clusters rather than by a predefined numerical target.

The sample included 536 boys (56.4%) and 404 girls (42.5%), while 10 participants (1.1%) had missing gender information. After excluding these cases, boys represented 57% and girls 43% of the analytical sample.

Regarding school type, 68% of participants (*n* = 639) attended public schools, whereas 32% (*n* = 301) were enrolled in private institutions. Additional sociodemographic characteristics and gender-based comparisons are presented in Supplementary Tables 1, 2. Although a statistically significant difference in age was observed between genders, the effect size was negligible (Cohen’s *d* = 0.163).

As previously indicated, the analytical sample comprised 940 adolescents aged 14–18 years with a higher proportion of males than females. Most participants attended public secondary schools and reported medium-to-high family affluence levels. The sample was predominantly from Spain, with smaller representations from other geographical regions. Detailed sociodemographic characteristics of the total sample are presented in Supplementary Table 1.

Gender-based comparisons of sociodemographic variables are reported in Supplementary Table 2. Results indicated a statistically significant difference in age between males and females. However, the associated effect size was small. No significant gender differences were observed for nationality, perceived socioeconomic status, or educational institution type, suggesting a largely homogeneous distribution of these variables across gender groups.

### 2.2 Procedure

The data were collected during the 2022–2023 academic year in a single, in-person session lasting approximately 60 min within the participating schools. The administration of the instruments and the resolution of students’ inquiries were conducted by trained researchers, with sessions meticulously scheduled to circumvent disruption to standard academic activities. The study involved 23 educational centers and 47 classrooms, encompassing both public and private.

The study was conducted in accordance with the principles outlined in the World Medical Association Declaration of Helsinki (21) and adhered to the Spanish legislation pertaining to biomedical research, data protection, confidentiality, non-discrimination, and gratuity. Ethical approval was obtained from the University of Alicante Research Ethics Committee (UA-2023-02-17), the Government Delegation for the National Plan on Drugs, the regional Department of Education, Research, Culture and Sports, and the administrations of the participating schools.

Informed consent was obtained from parents or legal guardians, detailing the purpose of the study, data confidentiality, voluntary participation, the right to withdraw at any time, and exclusive use of personal data for research purposes. Students aged 12 years or older were required to provide their own assent, employing language commensurate with age. All decisions regarding participation were duly respected, and no participation was compelled.

### 2.3 Instruments

The Binge-Watching Engagement and Symptoms Questionnaire (BWESQ) (22) is a 40-item instrument designed to assess both adaptive and problematic dimensions of binge-watching behavior: (1) “Engagement,” refers to the level of involvement or participation in a given activity, such as watching television; (2) “Positive Emotions,” includes feelings of joy, satisfaction, and contentment; (3) “Desire Savoring,” refers to the perception of time and attention while watching, characterized by immersion in the viewing experience and the tendency to focus intensely on the series; (4) “Pleasure Preservation,” includes behaviors aimed at maintaining viewing enjoyment, such as organizing time or postponing other activities in order to continue watching; (5) “Binge-Watching,” refers to the satisfaction derived from watching multiple episodes consecutively, often associated with repetitive and potentially compulsive viewing behavior; (6) “Dependency,” includes social self-exclusion and feelings of irritability or discomfort when viewing is interrupted or not possible; and (7) “Loss of Control,” refers to the inability to reduce viewing time and the neglect of personal, academic, or occupational responsibilities. Responses were recorded on a four-point Likert scale, ranging from “strongly disagree” to “strongly agree.”

In regard to the psychometric properties of the instrument, Flayelle et al. reported excellent internal consistency for the general factor of the BWESQ (22) (*α* = 0.970, *ω* = 0.970) and acceptable to good reliability for the dimensions (*α* = 0.630–0.830). In the present study, the internal consistency for the general factor was also excellent (*α* = 0.948, *ω* = 0.960). Dimension-specific coefficients demonstrated comparable robustness, indicating a multidimensional structure for the instrument: Engagement (*α* = 0.900, *ω* = 0.900), Positive Emotions (*α* = 0.860, *ω* = 0.860), Desire Savoring (*α* = 0.870, *ω* = 0.870), Pleasure Preservation (*α* = 0.800, *ω* = 0.870), Binge-Watching (*α* = 0.900, *ω* = 0.900), Dependency (*α* = 0.860, *ω* = 0.860), and Loss of Control (*α* = 0.930, *ω* = 0.930), supporting the multidimensional structure and reliability of the instrument.

The Teen Executive Functioning Inventory (TEXI) was employed to assess EF (23). This instrument was composed of 20 items, which were divided into two primary dimensions: (1) first, Inhibition reflected the capacity to suppress automatic responses; whereas (2) Working Memory captured the ability to actively maintain and manipulate information. The TEXI is scored on a 5-point Likert scale ranging from 1 (“definitely not true”) to 5 (“definitely true”), with higher scores indicating greater difficulties in executive functioning. Accordingly, higher scores reflect poorer inhibitory control and working memory performance (23). Costa-López et al. conducted a Spanish validation that demonstrated good reliability values for the general factor of EF (24) (Cronbach’s *α* = 0.946, McDonald’s *ω* = 0.947), as well as for the inhibition dimension (*α* = 0.905, *ω* = 0.906) and the working memory dimension (*α* = 0.905, *ω* = 0.905). In the present study sample, reliability was similarly robust, with the general factor showing *α* = 0.949 and *ω* = 0.949, inhibition *α* = 0.906 and *ω* = 0.906, and working memory *α* = 0.916 and *ω* = 0.916.

The Family Affluence Scale (FAS) (25) was a brief self-report measure of adolescent family socioeconomic status, based on concrete indicators of material wealth. It included six items, each assessed on a categorical scale, which addressed the following aspects of households’ economic and material resources: the number of cars, computers, bathrooms, ownership of a bedroom, family vacations, and ownership of a dishwasher. Each item was scored on a categorical scale and summed to create a total score.

In the present study, the Family Affluence Scale (FAS) was used as a covariate to control for socioeconomic differences between participants. Internal consistency was low (Cronbach’s *α* = 0.497; McDonald’s *ω* = 0.537), which is expected given the formative nature of the scale and the heterogeneous content of its items. Previous studies using adolescent samples have similarly reported modest internal consistency estimates for the FAS, supporting the interpretation that lower reliability indices are common for this type of socioeconomic indicator (26–28). Accordingly, reliability indices based on internal consistency should be interpreted with caution (28–30). Although the FAS was included as a covariate to account for socioeconomic differences, the potential influence of measurement error derived from its heterogeneous indicators should be considered when interpreting adjusted associations.

For the analyses, the total FAS score was used to categorize participants into three family affluence groups following standard Health Behavior in School-aged Children (HBSC) (World Health Organization Regional Office for Europe, 2020) procedures: low (0-6), medium (7-9), and high (10-13) affluence.

### 2.4 Statistical analysis

Preliminary inspection of the data distribution was conducted using skewness and kurtosis indices, with values falling within acceptable ranges (skewness ±2; kurtosis ±7) (31–33). Additionally, the large sample size (*N* = 940) supports the robustness of parametric analyses under the assumptions of the Central Limit Theorem. Values remained within acceptable ranges, supporting the use of parametric analyses despite minor deviations from normality (34, 35). Formal normality tests were not prioritized due to their sensitivity in large samples (36).

Descriptive statistics (means, standard deviations, and ranges) were computed for all variables. Gender differences were examined using independent samples *t*-tests, with effect sizes reported using Cohen’s *d*.

Associations between BW (BWESQ) and EF (TEXI) were examined using partial Pearson correlations, controlling for age, gender and Family Affluence Scale (FAS). Analyses were conducted for the total sample and by gender, with 95% confidence intervals estimated for all coefficients. Given the formative nature of the FAS, its inclusion as a covariate was intended to account for potential socioeconomic differences rather than to provide a latent estimate of socioeconomic status. A sensitivity power analysis was conducted for the partial correlation analyses. With *N* = 940, three covariates, a two-tailed *α* level of 0.05, and 80% statistical power, the minimum detectable partial correlation was |r| = 0.091, indicating adequate sensitivity to detect small associations in the total sample.

To account for multiple testing in the partial correlation analyses, *p*-values were adjusted using the False Discovery Rate (FDR) approach with the Benjamini-Hochberg procedure. This correction was applied to the set of partial correlations examining associations between BWESQ and TEXI dimensions. The corrected *p*-values were used to determine statistical significance, while the original correlation coefficients and 95% confidence intervals were retained for interpretation of effect size and precision. All associations that were statistically significant before correction remained significant after FDR adjustment.

Network analyses were conducted to model multivariate associations. Networks were estimated as Gaussian Graphical Models (GGMs), where edges represent regularized partial correlations reflecting conditional dependencies.

To improve sparsity and interpretability, networks were estimated using the graphical least absolute shrinkage and selection operator (Glasso), in line with current recommendations for psychological network estimation (37, 38). Model selection was based on the Extended Bayesian Information Criterion (EBIC; *γ* = 0.25). A moderate regularization parameter was selected to balance network sparsity and the retention of potentially meaningful associations (37).

Variables were treated as approximately continuous following standard practice in psychological network analysis with Likert-type indicators, and nodes corresponded to the mean scores of each BWESQ and TEXI dimension.

Analyses were conducted using JASP (version 0.19.3.0) (39), SPSS (version 29.0) (40), and R (version 4.5.2) (41). Network analyses were performed in R using the packages qgraph, bootnet, EGAnet, and NetworkComparisonTest.

Network accuracy was assessed via non-parametric bootstrap resampling (1,000 iterations), providing 95% confidence intervals for edge estimates. Centrality stability was evaluated using a case-dropping bootstrap procedure (Correlation Stability coefficient).

Gender differences were tested using the Network Comparison Test (NCT) with 1,000 permutations, assessing both network structure invariance and global strength invariance.

Exploratory Graph Analysis (EGA) was used to identify community structure.

All tests were two-tailed with *p* < 0.05.

## 3 Results

### 3.1 Preliminary analyses: descriptive statistics and gender differences on the study variables

Descriptive statistics for the binge-watching (BWESQ) and executive functioning (TEXI) dimensions are presented in Tables 1, 2, including means, standard deviations, and observed ranges.

**Table 1:** Descriptive statistics for BWESQ dimensions and gender comparisons.

BWESQ’s dimensions	Total sample; *n* = 940 *M* (SD)	Males; *n* = 536 *M* (SD)	Females; *n* = 404 *M* (SD)	Min	Max	Skewness (total)	Kurtosis (total)	Skewness (males)	Kurtosis (males)	Skewness (females)	Kurtosis (females)	*p*-value	Cohen’s *d*
Engagement	15.47 (5.61)	14.95 (5.54)	16.17 (5.64)	8.00	32.00	0.471	−0.315	0.577	−0.108	0.344	−0.479	<0.001^*^	−0.219
Positive emotions	10.29 (3.81)	9.87 (3.75)	10.83 (3.83)	5.00	20.00	0.232	−0.697	0.300	−0.696	0.142	−0.663	<0.001^*^	−0.256
Desire savoring	13.32 (4.59)	12.70 (4.64)	14.13 (4.39)	6.00	24.00	0.031	−0.661	0.152	−0.719	−0.091	−0.452	<0.001^*^	−0.315
Pleasure preservation	6.08 (2.43)	5.94 (2.44)	6.27 (2.40)	3.00	12.00	0.367	−0.653	0.432	−0.631	0.293	−0.642	0.035^*^	−0.139
Binge-watching	10.91 (4.32)	10.67 (4.27)	11.23 (4.37)	6.00	24.00	0.678	−0.005	0.670	−0.092	0.688	0.092	0.048^*^	−0.130
Dependency	8.76 (3.41)	8.60 (3.43)	8.97 (3.37)	5.00	20.00	0.860	0.516	0.877	0.503	0.854	0.578	0.104	−0.107
Loss of control	11.63 (4.83)	11.59 (4.88)	11.69 (4.78)	7.00	28.00	0.966	0.535	0.998	0.641	0.927	0.406	0.767	−0.019
General factor	76.46 (26.03)	74.33 (26.26)	79.30 (25.46)	40.00	160.00	0.511	0.097	0.582	0.199	0.447	0.046	0.004^*^	−0.192

*M*, mean; SD, standard deviation. Skewness and kurtosis values are reported separately for the total sample and by gender. *p*-values correspond to independent samples *t*-test comparing males and females. Cohen’s *d* represents effect size. Asterisks (^*^) indicate statistical significance at *p* < 0.05. Min, minimum; Max, maximum.

**Table 2:** Descriptive statistics for TEXI dimensions and gender comparisons.

TEXI’s dimensions	Total sample; *n* = 940 *M* (SD)	Males; *n* = 536 *M* (SD)	Females; *n* = 404 *M* (SD)	Min	Max	Skewness (total)	Kurtosis (total)	Skewness (males)	Kurtosis (males)	Skewness (females)	Kurtosis (females)	*p*-value	Cohen’s *d*
Inhibition	2.43 (0.83)	2.38 (0.81)	2.50 (0.85)	1.00	5.00	0.126	−0.064	0.131	0.081	0.100	−0.221	0.042^*^	−0.134
Working memory	2.30 (0.85)	2.23 (0.82)	2.40 (0.87)	1.00	5.00	0.491	0.239	0.597	0.692	0.350	−0.165	0.002^*^	−0.204
General factor	2.37 (0.80)	2.31 (0.78)	2.45 (0.83)	1.00	5.00	0.238	0.122	0.310	0.451	0.133	−0.182	0.009^*^	−0.173

*M*, mean; SD, standard deviation. Skewness and kurtosis values are reported separately for the total sample and by gender. *p*-values correspond to independent samples *t*-test comparing males and females. Cohen’s *d* represents effect size. Asterisks (^*^) indicate statistical significance at *p* < 0.05. Min, minimum; Max, maximum.

Gender differences were examined using independent samples *t*-tests, with effect sizes reported using Cohen’s *d*.

### 3.2 Relationship between binge-watching and executive functioning

Partial Pearson correlations were conducted to examine the relationship between binge-watching behaviors and executive functioning while controlling for covariates (gender, age and FAS). Analyses were performed for the total sample and separately by gender to explore potential subgroup-specific patterns (Table 3).

**Table 3:** Partial correlation matrix among BWESQ and TEXI dimensions (controlling for age, gender, and FAS) with 95% confidence intervals (*n* = 940).

Dimension	TEXI (In)	TEXI (WM)	TEXI (GF)	BWESQ (E)	BWESQ (PE)	BWESQ (DS)	BWESQ (PP)	BWESQ (BW)	BWESQ (De)	BWESQ (LC)	BWESQ (GF)
TEXI (In)	–										
TEXI (WM)	0.839 [0.814, 0.862]^***^	–									
TEXI (GF)	0.966 [0.961, 0.971]^***^	0.951 [0.942, 0.958]^***^	–								
BWESQ (E)	0.370 [0.300, 0.432]^***^	0.365 [0.297, 0.430]^***^	0.383 [0.312, 0.447]^***^	–							
BWESQ (PE)	0.395 [0.330, 0.456]^***^	0.378 [0.318, 0.443]^***^	0.403 [0.343, 0.466]^***^	0.862 [0.842, 0.880]^***^	–						
BWESQ (DS)	0.402 [0.342, 0.458]^***^	0.387 [0.324, 0.443]^***^	0.412 [0.349, 0.471]^***^	0.795 [0.768, 0.820]^***^	0.813 [0.786, 0.837]^***^	–					
BWESQ (PP)	0.356 [0.291, 0.418]^***^	0.345 [0.276, 0.413]^***^	0.366 [0.304, 0.433]^***^	0.751 [0.715, 0.784]^***^	0.763 [0.723, 0.798]^***^	0.710 [0.671, 0.747]^***^	–				
BWESQ (BW)	0.347 [0.278, 0.407]^***^	0.367 [0.299, 0.432]^***^	0.371 [0.304, 0.433]^***^	0.860 [0.840, 0.881]^***^	0.791 [0.762, 0.818]^***^	0.734 [0.701, 0.764]^***^	0.696 [0.654, 0.734]^***^	–			
BWESQ (De)	0.348 [0.275, 0.413]^***^	0.370 [0.303, 0.437]^***^	0.373 [0.305, 0.439]^***^	0.813 [0.784, 0.839]^***^	0.730 [0.693, 0.763]^***^	0.678 [0.639, 0.711]^***^	0.667 [0.623, 0.708]^***^	0.871 [0.848, 0.893]^***^	–		
BWESQ (LC)	0.303 [0.232, 0.367]^***^	0.346 [0.277, 0.414]^***^	0.337 [0.268, 0.400]^***^	0.797 [0.766, 0.827]^***^	0.679 [0.638, 0.717]^***^	0.593 [0.546, 0.634]^***^	0.612 [0.562, 0.658]^***^	0.878 [0.855, 0.900]^***^	0.892 [0.870, 0.910]^***^	–	
BWESQ (GF)	0.401 [0.331, 0.462]^***^	0.408 [0.341, 0.471]^***^	0.421 [0.354, 0.482]^***^	0.949 [0.942, 0.956]^***^	0.899 [0.885, 0.912]^***^	0.853 [0.833, 0.869]^***^	0.808 [0.780, 0.833]^***^	0.939 [0.929, 0.947]^***^	0.905 [0.890, 0.920]^***^	0.882 [0.862, 0.898]^***^	–

Partial Pearson correlations controlling for gender, age and Family Affluence Scale (FAS) are reported. Values in brackets represent 95% confidence intervals. All correlations are two-tailed. *p*-values were adjusted for multiple comparisons using the False Discovery Rate (FDR) procedure with the Benjamini-Hochberg ^***^indicates statistical significance at FDR-adjusted *p* < 0.001. TEXI (In), Inhibition, TEXI (WM), Working Memory, TEXI (GF), General Factor. BWESQ (E), Engagement, BWESQ (PE), Positive Emotions, BWESQ (DS), Desire Savoring, BWESQ (PP), Pleasure Preservation, BWESQ (BW), Binge-Watching, BWESQ (De), Dependency, BWESQ (LC), Loss of Control, BWESQ (GF), General Factor.

Consistently with the coding of the executive inventory, partial Pearson correlations showed positive associations between BWESQ and TEXI dimensions after controlling for gender, age, and FAS. Overall, greater executive functioning difficulties were associated with higher levels of engagement-related and problematic binge-watching dimensions. Associations were generally moderate, with the strongest relationships observed between the BWESQ General Factor and TEXI dimensions. Complete partial correlation coefficients and 95% confidence intervals are presented in Table 3.

### 3.3 Network analysis for total sample

The global interplay between binge-watching behaviors (BWESQ) and executive functioning (TEXI) was first modeled using network analysis to elucidate the organizational structure of the constructs, providing an overall representation of the associations between behavioral and cognitive dimensions before exploring gender-specific comparisons.

The network comprised nine nodes, corresponding to the seven BWESQ dimensions (Engagement, Positive Emotions, Desire Savoring, Pleasure Preservation, Binge- Watching, Dependency, and Loss of Control) and the two TEXI dimensions (Inhibition and Working Memory). Of the 36 possible edges, 27 were non-zero, yielding a sparsity level of 0.25. The resulting network structure is displayed in Figure 1.

**Figure 1:**
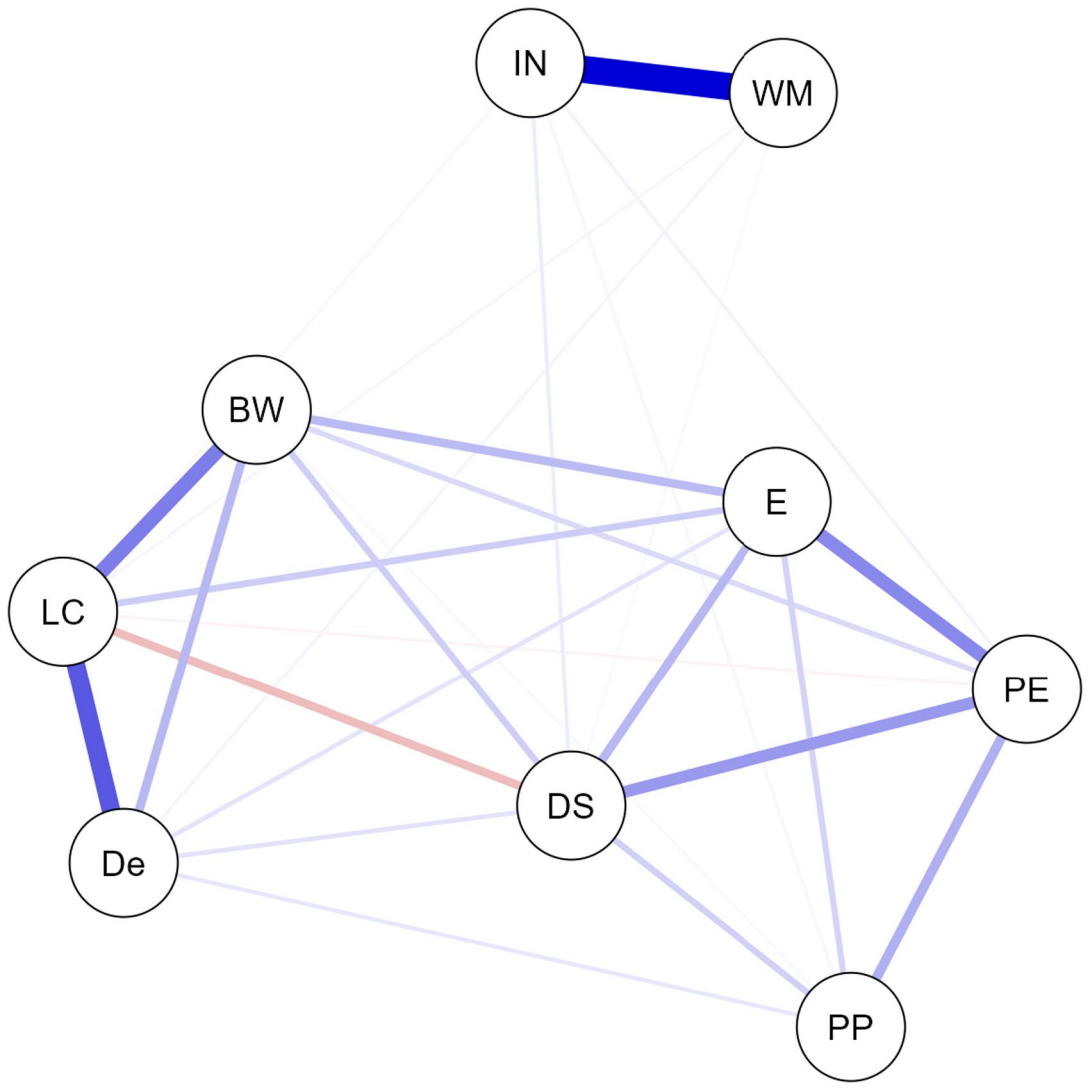
Network graph for the total sample. E, Engagement; PE, Positive Emotions; DS, Desire Savoring; PP, Pleasure Preservation; BW, Binge-Watching; De, Dependency; LC, Loss of Control; IN, Inhibition; WM, Working Memory. Edge thickness reflects the magnitude of partial correlations; red edges indicate negative associations.

The estimated network showed strong associations among BWESQ dimensions, particularly between Binge-Watching, Dependency, and Loss of Control. Executive functioning nodes were strongly interconnected, with a prominent association between Inhibition and Working Memory. Connections between BWESQ and TEXI dimensions were present but comparatively weaker.

Node centrality was assessed using strength, expected influence (EI), closeness, and betweenness indices (Supplementary Table 3). Among BWESQ dimensions, Engagement and Binge-Watching exhibited the highest expected influence values, followed by Positive Emotions. Pleasure Preservation showed the lowest centrality across indices. Inhibition and Working Memory displayed lower strength and expected influence relative to binge-watching dimensions.

Normalized expected influence estimates for all nodes are shown in Supplementary Figure 1.

The robustness of the estimated networks was evaluated through two distinct bootstrap procedures using the bootnet package. First, edge-weight accuracy was assessed via non-parametric bootstrap analyses (1.000 iterations). As shown in Supplementary Figure 2, the narrow 95% confidence intervals (Cis) around the estimated edge weights, particularly for the strongest associations like the Inhibition-Working Memory (IN–WM) pathway, indicate high precision in parameter estimation.

Second, the stability of node centrality was examined using a case-dropping bootstrap procedure. The Correlation Stability (CS) coefficient for strength centrality was 0.67, which well exceeds the recommended threshold of 0.50 (37). This confirms that the order of node importance remains stable even after dropping 67% of the original sample. These results, which were replicated across gender-specific networks, justify the subsequent interpretation of network topology and the comparison of global strength and structure.

Exploratory Graph Analysis identified three communities within the total sample network. The first community included Engagement, Positive Emotions, Desire Savoring, and Pleasure Preservation. The second comprised Binge-Watching, Dependency, and Loss of Control, and the third consisted of Inhibition and Working Memory.

Community stability was high, with replication values close to equal to 1 for all nodes (PE, DS, PP, IN, WM = 1, E = 0.99, BW = 0.99, De = 0.98, LC = 0.98). The EGA community structure is displayed in Figure 2.

**Figure 2:**
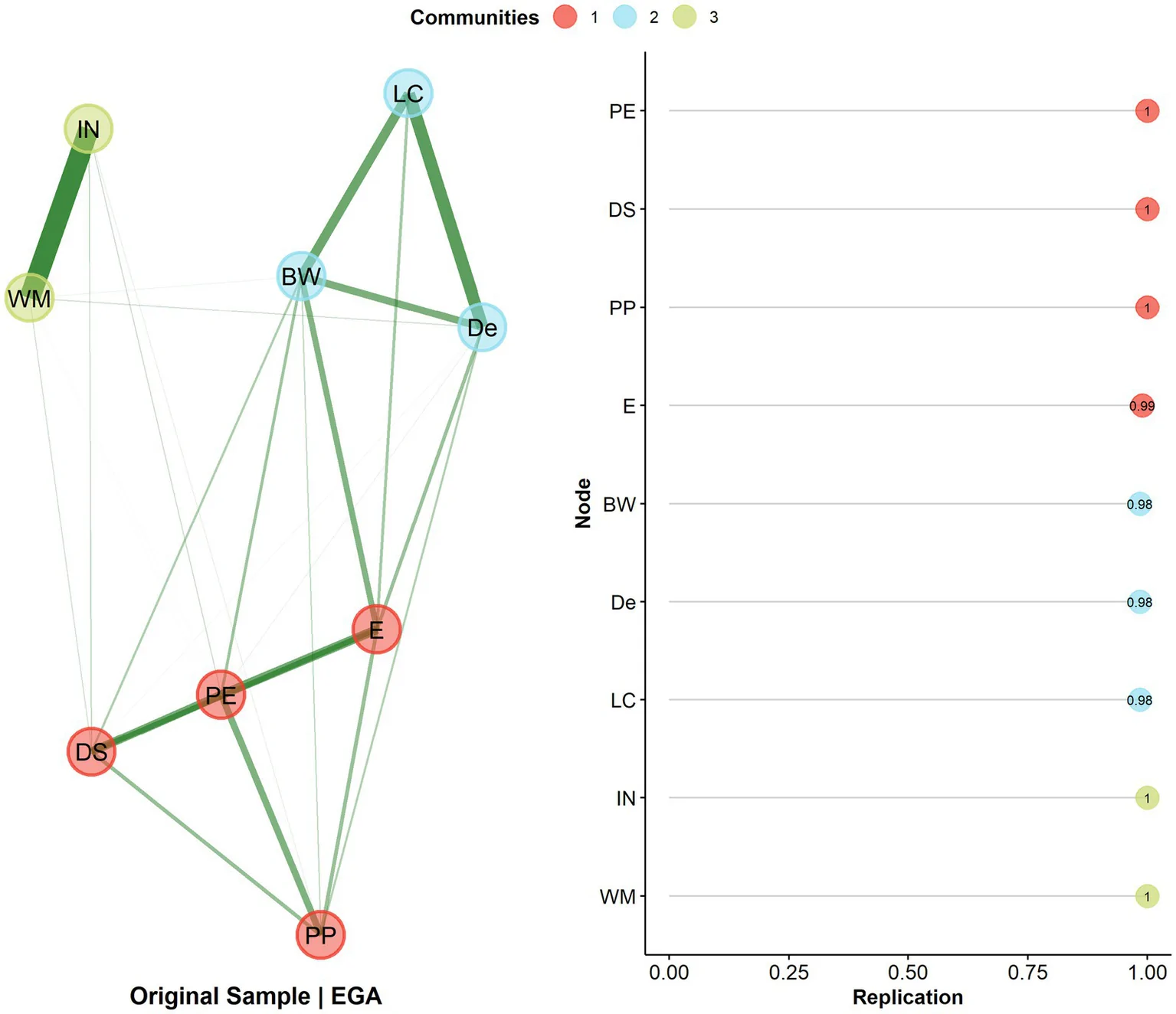
EGA community structure for total sample. Nodes are colored by community: Red = Engagement cluster (E, Engagement, DS, Desire Savoring, PE, Positive Emotions, PP, Pleasure Preservation), blue, Binge-Watching cluster (BW, Binge-Watching, De, Dependency, LC, Loss of Control), green, Executive Functioning cluster (IN, Inhibition, WM, Working Memory).

Bridge Expected Influence (Bridge EI) was not estimated for the total sample network, reflected by a large proportion of non-zero edges, which limits the interpretability of bridge metrics, as nodes display extensive connectivity both within and across communities. Under such conditions, bridge estimates may be inflated or conceptually ambiguous. In contrast, gender-specific networks were less dense and exhibited clearer modular organization, allowing for a more meaningful identification of nodes facilitating inter-community connectivity. Accordingly, Bridge EI analyses were conducted exclusively within gender-stratified networks, where community boundaries were more clearly defined and bridging roles could be interpreted with greater precision.

### 3.4 Gender-specific network analysis

The interplay between BW behaviors (BWESQ) and EF (TEXI) was examined using network analysis, separately for men and women.

Both networks comprised nine nodes. In men, 27 of 36 possible edges were non-zero (sparsity = 0.25), whereas in women, 26 edges were non-zero (sparsity = 0.278). The strongest associations were observed among TEXI dimensions, with moderate connections between BWESQ dimensions and a small inverse association between Loss of Control and Desire Savoring (Figures 3, 4).

**Figure 3:**
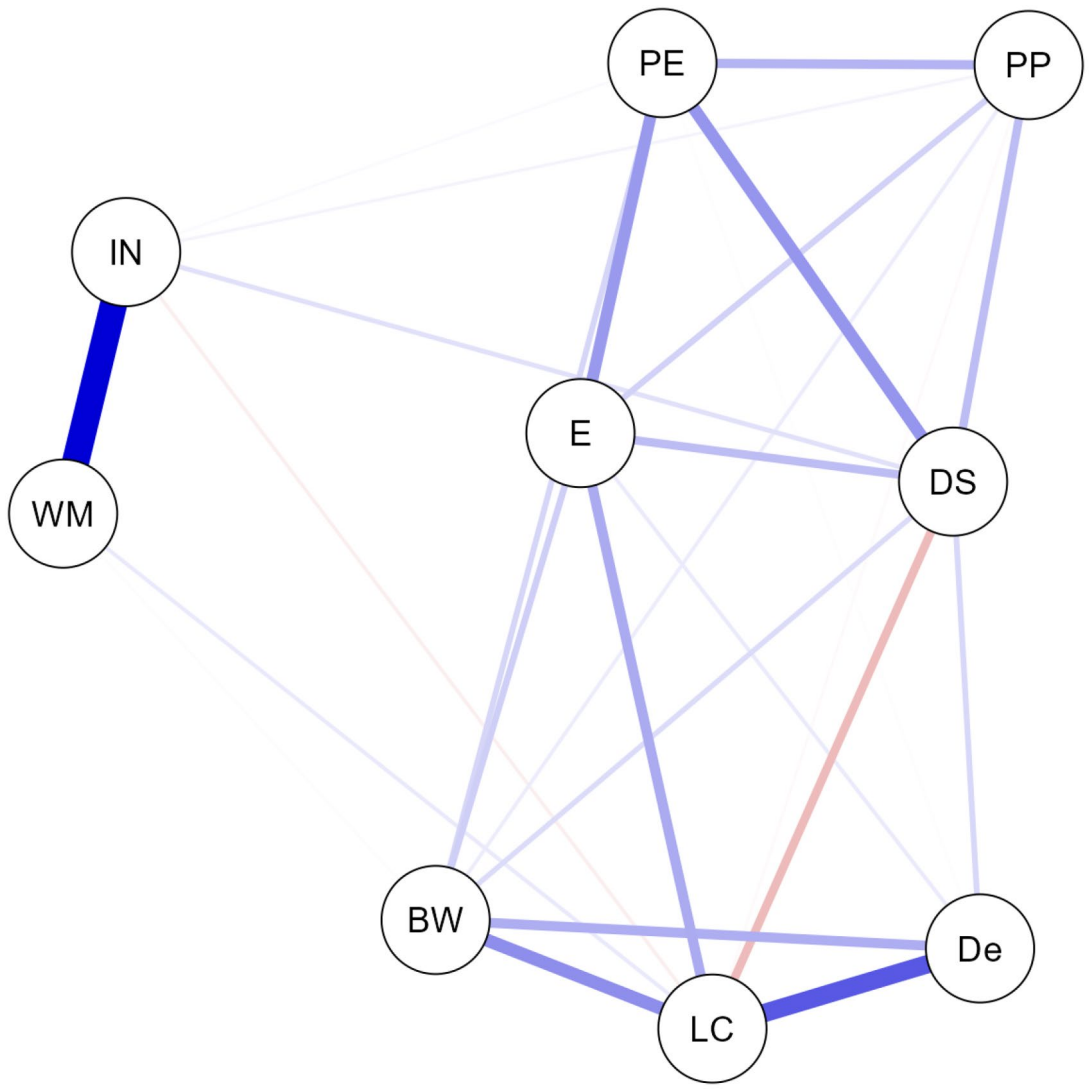
Network graph for men. E, engagement; PE, positive emotions; DS, desire savoring; PP, pleasure preservation; BW, binge-watching; De, dependency; LC, loss of control; IN, inhibition; WM, working memory. Red edges indicate negative associations.

**Figure 4:**
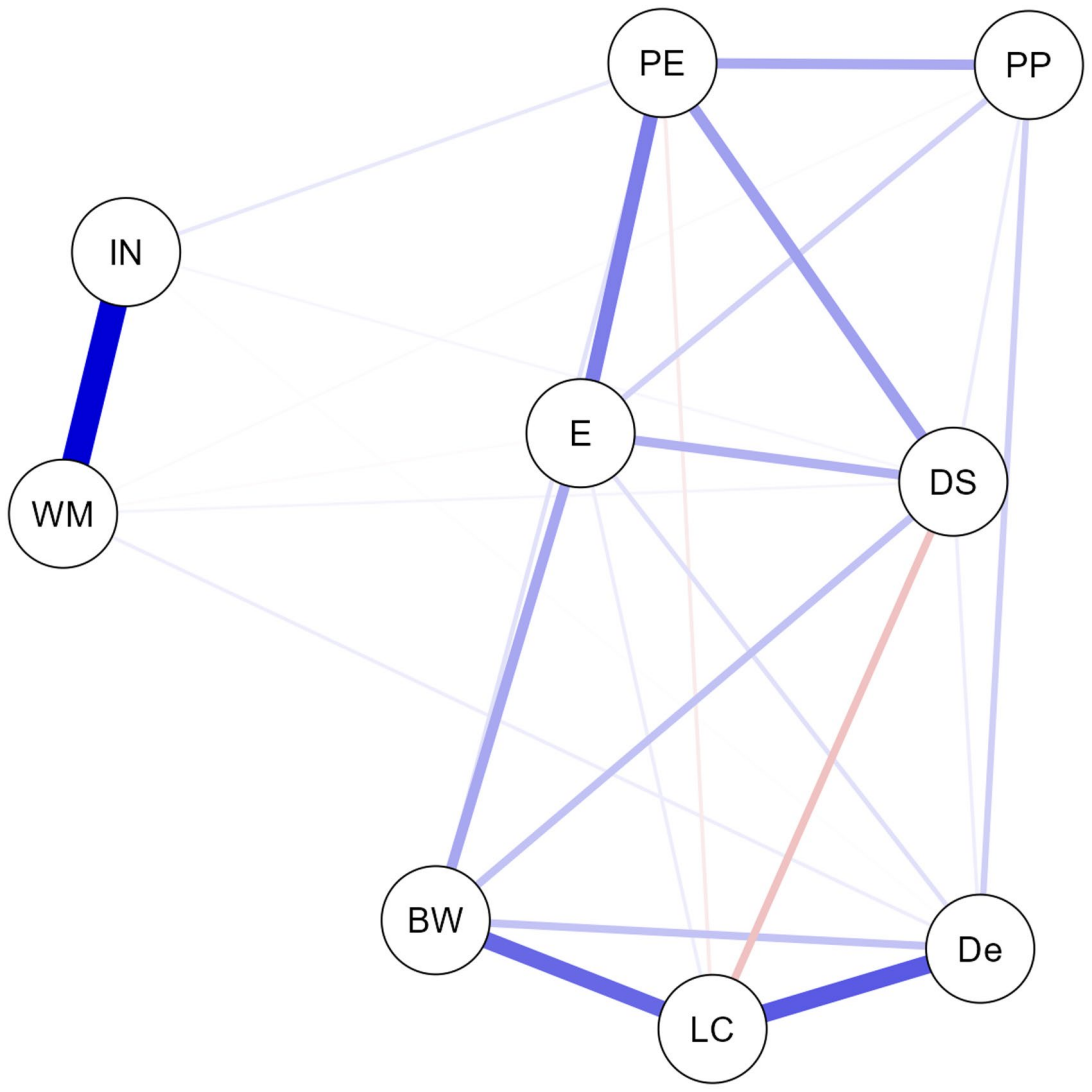
Network graph for women. E, engagement, PE, positive emotions; DS, desire savoring; PP, pleasure preservation; BW, binge-watching, De, dependency; LC, loss of control; IN, inhibition; WM, working memory. Red edges indicate negative associations.

In this study, edge weights were highly stable (CS = 0.75), and strength centrality was acceptable (CS = 0.51), supporting robust network estimation. Node-level variance explained by connections was moderate (*R*^2^ = 0.401 for men; *R*^2^ = 0.456 for women), with slightly stronger interrelations in the female network. In men, Engagement, Binge-Watching, and Positive Emotions exhibited the highest Expected Influence and Strength; in women, Binge-Watching, Engagement, and Desire Savoring dominated these metrics. Node-level Expected Influence estimates by gender are illustrated in the (Supplementary Figures 8, 9).

Bridge Expected Influence (Bridge EI) highlighted nodes facilitating inter-community communication. In men, Binge-Watching, Engagement, and Dependency showed the highest Bridge EI values, whereas in women, Binge-Watching, Engagement, Dependency, and Desire Savoring connected communities. Notably, Loss of Control showed negligible bridge expected influence values in all genders (Bridge EI = 0.000; Table 4), suggesting that although this dimension reflects problematic binge-watching characteristics, it did not function as a connecting node between the identified communities within the estimated network structure. Betweenness and closeness were calculated but are less reliable in small networks and are described only narratively (Figures 5, 6). Bridge Expected Influence estimates for each node and gender are provided in the (Supplementary Figures 5, 8, 9).

**Table 4:** Expected influence and bridge expected influence by node and gender.

Variable	EI-Men	EI-Women	Bridge EI-Men	Bridge EI-Women
Engagement (BWESQ)	1.151	1.174	1.000	0.805
Positive emotions (BWESQ)	1.039	1.045	0.231	0.390
Desire savoring (BWESQ)	0.862	0.694	0.143	0.406
Pleasure preservation (BWESQ)	0.665	0.615	0.063	0.475
Binge-watching (BWESQ)	1.067	1.195	0.942	1.000
Dependency (BWESQ)	0.976	1.036	0.335	0.730
Loss of control (BWESQ)	0.932	0.778	0.000	0.000
Inhibition (TEXI)	0.897	0.880	0.097	0.392
Working memory (TEXI)	0.879	0.867	0.055	0.375

EI, Expected Influence; Bridge EI, Bridge Expected Influence. Values are reported separately for men and women to facilitate comparison across gender-specific networks. Higher values indicate greater node influence or greater bridging capacity between communities.

**Figure 5:**
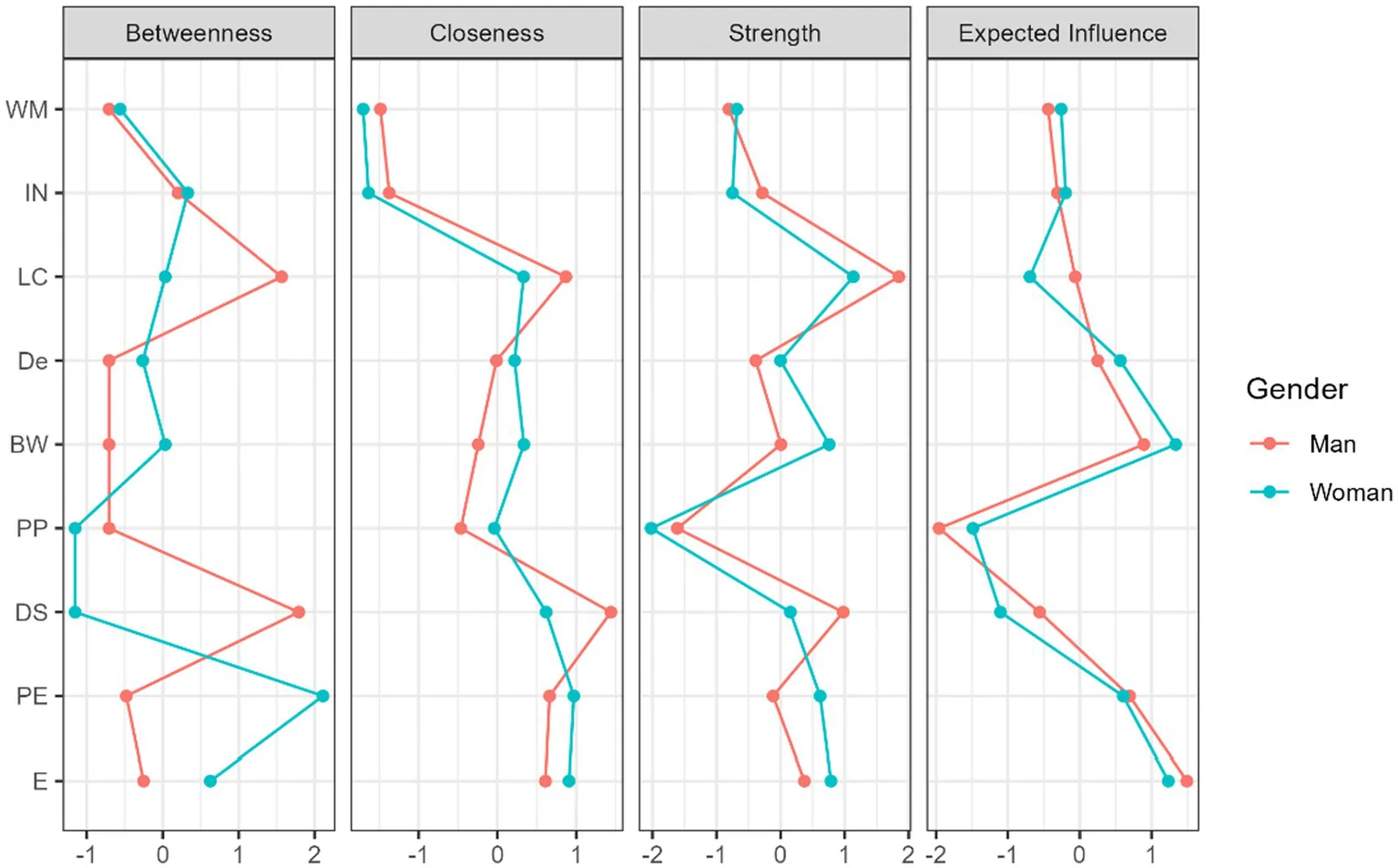
Node centrality indices by gender. E, engagement, PE, positive emotions; DS, desire savoring; PP, pleasure preservation; BW, binge-watching, De, dependency; LC, loss of control; IN, inhibition; WM, working memory. Centrality estimates are displayed separately by gender, with red lines corresponding to men and blue lines corresponding to women.

**Figure 6:**
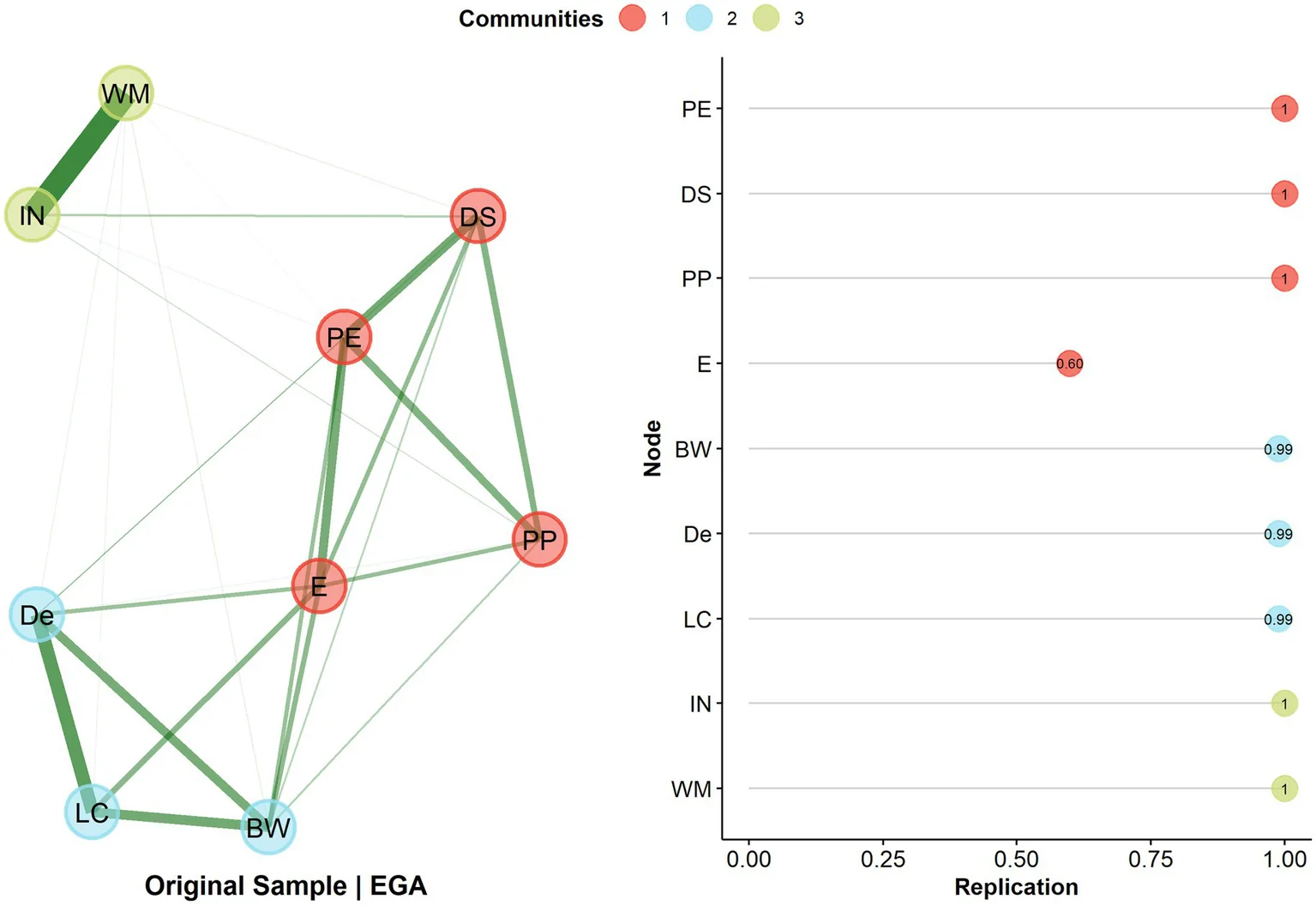
EGA community structure for men. Nodes are colored by community: Red, Engagement cluster (E, Engagement, DS, Desire Savoring, PE, Positive Emotions, PP, Pleasure Preservation), blue, Binge-Watching cluster (BW, Binge-Watching, De, Dependency, LC, Loss of Control), green, Executive Functioning cluster (IN, Inhibition, WM, Working Memory).

Exploratory Graph Analysis (EGA) revealed three highly stable communities in all genders. In men, the first community included Engagement, Desire Savoring, and Positive Emotions (replication ≈ 0.58, for Engagement, ≈1 for the others), the second encompassed Binge-Watching and related dimensions (replication ≈ 0.98), and the third comprised TEXI dimensions (replication = 1). In women, replication indices approached 1 across all nodes, indicating extremely high stability and modularity (Figures 6, 7).

**Figure 7:**
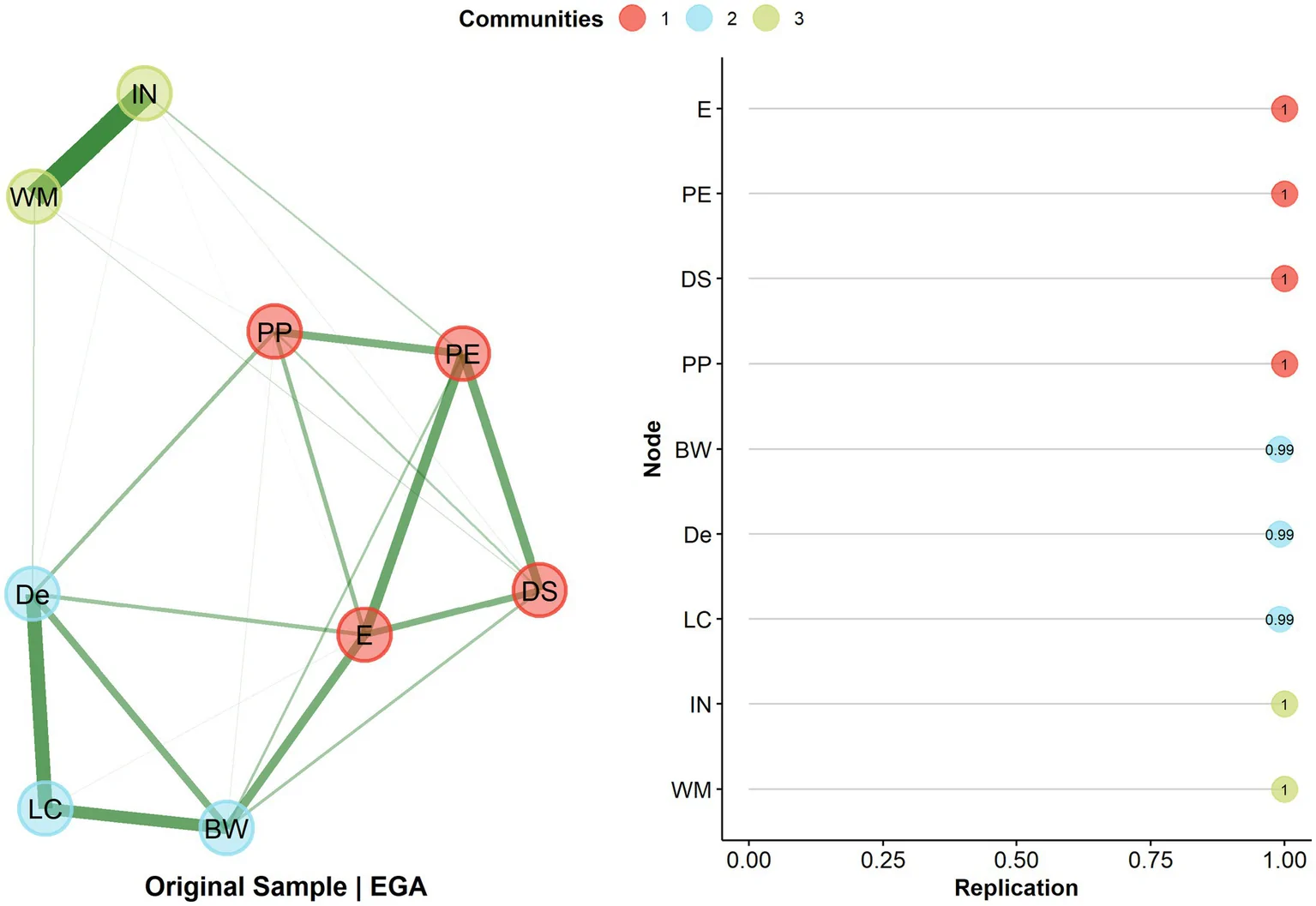
EGA community structure for women. Nodes are colored by community: red, Engagement cluster (E, Engagement, DS, Desire Savoring, PE, Positive Emotions, PP, Pleasure Preservation), blue, Binge-Watching cluster (BW, Binge-Watching, De, Dependency, LC, Loss of Control), green, Executive Functioning cluster (IN, Inhibition, WM, Working Memory).

Gender differences were formally assessed using the Network Comparison Test (NCT). The Network Invariance Test revealed a significant difference in the overall structure (*M* = 0.213, *p* = 0.031), indicating that the configuration of associations between binge-watching and executive functions is gender-dependent. In contrast, Global Strength Invariance was non-significant (*S* = 0.129, *p* = 0.559), confirming that while the “shape” of the network architecture differs, the total intensity of interconnectedness remains comparable between genders. The Bridge Expected Influence (Bridge EI) and centrality patterns further reinforced these findings: Binge-Watching and Engagement were consistently central and bridging across both groups, while secondary nodes showed nuanced gender-specific modulation in their roles within the network. However, Loss of Control did not exhibit a bridging role in either gender, suggesting that its contribution may be more closely related to the internal structure of the problematic binge-watching community rather than inter-community connectivity.

## 4 Discussion

The present study examined the associations between BW dimensions and EF difficulties, particularly inhibition and working memory, using both correlational and network-based approaches, while also exploring gender differences in the network structure linking these constructs. In doing so, it offers a detailed characterization of the structural organization of binge-watching in adolescence from a network perspective.

The findings support a multidimensional conceptualization of BW rather than viewing it as an inherently addictive behavior, suggesting a tripartite organization in which engagement-related, dysregulated viewing, and cognitive control emerge as distinct yet interconnected functional communities.

A key finding is the clear segregation of the “Engagement” and “Binge-Watching” clusters. This mathematical modularity, supported by high community stability (replication values ≥ 0.98 for most nodes in the total-sample network), is consistent with the conceptual distinction proposed by Flayelle et al., in which healthy immersion is distinguished from dysregulated consumption (4, 22, 42, 43). The fact that Engagement and Desire Savoring form a separate community, distinct from Loss of Control suggests that intensive viewing in adolescence often reflects a “harmonious passion,” as conceptualized in the Dualistic Model of Passion, which differentiates harmonious engagement, driven by intrinsic motivation, from obsessive engagement, characterized by rigid and compulsive involvement rather than compulsive behavior (44, 45). These findings align with prior work cautioning against the over-pathologization of everyday behaviors (46, 47). Even in a high-exposure media context such as the Spanish adolescent environment (8), the psychological experience appears predominantly non-pathological. Structural independence may suggest that high-involvement nodes alone are not necessarily associated with functional impairment, whereas stronger connectivity involving bridge nodes such as Dependency may represent a network configuration more closely related to deregulated patterns.

Self-Determination Theory further suggests that autonomous motivation may support more adaptive forms of media use, whereas controlled motivation may be associated with more dysregulated patterns (48, 49). In adolescent BW, this framework may help explain why high involvement does not necessarily imply functional impairment. Rather than reflecting problematic use per se, engagement-related dimensions may represent voluntary and intrinsically motivated viewing experiences, whereas bridge nodes such as Dependency may indicate points of connection with more problematic patterns of BW behavior (50).

The role of Working Memory may be interpreted within a resource-demand framework, whereby sustained engagement with on-demand content may place demands on limited cognitive resources involved in attention, inhibition, and self-regulation (51–55). Previous research on cognitive load and media multitasking suggests that intensive engagement with multiple streams of information may be associated with reduced working memory performance and impaired control processes (51, 52, 56). From this perspective, BW may be interpreted within a resource-demand framework rather than as a mere failure of willpower (55, 57, 58). A CS-coefficient of 0.67 indicates that this hierarchical role of executive functions (EFs) is a stable feature of the estimated network structure. Although Working Memory was connected to binge-watching dimensions within the estimated networks, the present findings should not be interpreted as indicating a primary bridging role (53, 55).

Gender-specific network analyses revealed divergent patterns. In females, Binge-Watching and Engagement showed the highest Expected Influence values, whereas Desire Savoring also contributed to cross-community connectivity (Bridge EI = 0.406), suggesting a “bottom-up” pattern in which emotionally salient content may be associated a with greater engagement through affective processes (59, 60). From this perspective, emotional immersion may be linked to a higher subjective reward value of media consumption, promoting sustained viewing independently of executive control demands. This aligns with prior research on gendered media consumption and emotional regulation (61, 62), as well as more recent evidence indicating that adolescent girls show greater involvement in emotion-driven digital engagement and are more likely to use media for emotional regulation processes (63, 64).

In males, Inhibition showed a relatively high Expected Influence (EI = 0.897), indicating an influential position within the network. However, its Bridge Expected Influence was comparatively low (Bridge EI = 0.097). Thus, although inhibitory control appeared relatively central within the male network, it did not show a prominent role in connecting the identified communities. In contrast, Inhibition showed a higher Bridge Expected Influence in females (Bridge EI = 0.392), suggesting a comparatively greater role in terms of overall network influence and cross-community connectivity. Nevertheless, the theoretical relevance of inhibitory control remains consistent with previous research suggesting that gender differences in digital behavior may partly reflect differential developmental trajectories in self-regulation and risk-related processing, with boys showing greater impulsivity and sensitivity to reward-driven behaviors in online contexts (11, 65, 66). From this perspective, difficulties in inhibitory control may still be relevant to the regulation of rewarding digital behaviors, although the present network estimates do not support interpreting inhibition as a stronger bridging mechanism in males (12, 67, 68). Case-dropping bootstrap analyses confirmed the robustness of these gender-specific networks, with CS coefficients of 0.75 for edge and 0.51 for strength centrality. These structural divergences underscore the importance of tailoring interventions to gender-specific cognitive-behavioral patterns rather than assuming uniform pathways of BW engagement and dysregulation (69).

Importantly, the absence of a bridging role for Loss of Control in either gender should not be interpreted as indicating a lack of relevance of this dimension. Rather, these findings suggest that Loss of Control may operate primarily as part of the problematic binge-watching community without serving as a central connecting node linking different functional domains of the network.

Beyond its methodological strengths, this study offers an important conceptual contribution by integrating BW with EF, two domains that have been largely examined in isolation. By linking patterns of media engagement with core cognitive control processes, the present findings extend models of BW beyond purely behavioral or addiction-based framework and situate it within a broader self-regulatory perspective. This integrative approach provides a more comprehensive understanding of adolescent media use, highlighting the potential role of cognitive mechanisms in shaping both adaptive and maladaptive engagement patterns.

This study benefits from high statistical power and rigorous bootstrap procedures, ensuring stable network estimates. Although the cross-sectional design precludes causal inferences, this approach is consistent with a growing body of research applying network analysis to examine psychological constructs at a structural level, where the primary aim is to identify patterns of associations rather than establish directionality (37, 70). Importantly, the magnitude of network edge weights should be interpreted as the relative strength of associations within the estimated network rather than as direct indicators of clinical significance. Given the cross-sectional and non-clinical nature of the present study, these associations do not have a direct clinical interpretation. Future longitudinal and clinically informed studies are needed to determine whether specific network connections are associated with meaningful functional impairment or clinically relevant outcomes.

However, several limitations should be acknowledged. First, the cross-sectional design prevents conclusions about temporal or causal relationships. Second, all measures were based on self-report questionnaires, which may be influenced by common-method variance and recall-bias. The use of a single assessment method may increase shared measurement-related variance among constructs, potentially affecting the strength of observed associations. Furthermore, retrospective reports of binge-watching behaviors may be affected by inaccuracies in recall or differences in perceived media use. Future research integrating self-reported measures with objective indicators of digital consumption, such as platform usage data or digital phenotyping approaches, may provide a more comprehensive assessment of binge-watching behaviors. Residual confounding cannot be excluded, as other unmeasured factors may have contributed to the observed associations.

Third, the absence of objective indicators of media consumption (e.g., platform usage data) limits the ability to validate self-reported binge-watching behaviors. In addition, total screen time was not assessed, limiting the possibility of distinguishing binge-watching patterns from broader patterns of overall screen exposure. Differences in the characteristics and affordances of streaming platforms were also not examined and may contribute to differences in viewing patterns.

Furthermore, psychiatric and psychological factors such as attention-deficit/hyperactivity disorder, depression, and anxiety were not assessed or controlled for and may represent additional sources of unmeasured confounding.

Finally, the generalizability of the findings may be constrained by the specific characteristics of the sample, and caution is warranted when extending these results to other populations or cultural contexts. Additionally, although the modest internal consistency of the FAS is expected for asset-based indices, the potential impact of measurement error should be considered when interpreting findings in which socioeconomic status was included as a covariate (71, 72).

Future research should incorporate digital phenotyping and objective platform usage logs to validate these subjective-cognitive mappings by integrating self-reported measures with real-time behavioral data, thereby reducing recall biases and enhancing the ecological validity of binge-watching assessment (73). Overall, these findings may inform prevention strategies that focus on environmental restructuring, targeting environmental cues potentially associated with reduced inhibitory control, rather than pathologizing the healthy engagement characteristic of modern adolescent media consumption (74).

This study advances current understanding of adolescent BW by applying network analysis to examine the structural interplay between binge-watching dimensions and executive functioning. The findings suggest that BW is better understood as a multidimensional relational system in which engagement, dysregulation, and cognitive control are organized distinctly by interconnected patterns of associations, rather than as a unitary or uniformly problematic behavior.

Importantly, the results reinforce the distinction between intensive yet non-pathological engagement and more dysregulated viewing patterns, highlighting the importance of avoiding the over-pathologization of common digital behaviors. Within this network structure, Working Memory emerged as a relevant connection point between executive functioning and binge-watching dimensions, while gender-specific configurations suggest differences in the cognitive and emotional organization of BW across adolescents.

Overall, these findings demonstrate the value of network analysis for providing a nuanced and non-pathologizing account of adolescent media consumption and may contribute to the development of more tailored prevention frameworks.

## Data Availability

The raw data supporting the conclusions of this article will be made available by the authors, without undue reservation.
