# Supplementary material for: Executive functioning and binge-watching behaviors in adolescents: a network analysis
Source: Front Public Health. 2026 Sep 29;14:1933368. doi: 10.3389/fpubh.2026.1933368 (PMC13658072; doi:10.3389/fpubh.2026.1933368)
Supplement: Supplementary file 1 [file Supplementary_file_1.docx]

***Supplementary Material***

**Supplementary Table 1**

*Sociodemographic information of the study sample (N= 940)*

| Variable | n(%)/M(SD) |
| --- | --- |
| Gender |  |
| - Male | 536 (57.0) |
| - Female | 404 (43.0) |
| Age |  |
| - Total | 15.44 (0.77) |
| - Male | 15.50 (0.80) |
| - Female | 15.37 (0.72) |
| Nationality |  |
| - Spanish | 823 (87.6) |
| - America (Central America and South America) | 67 (7.1) |
| - European countries | 21 (2.2) |
| - Africa | 20 (2.1) |
| - Asia | 9 (1.0) |
| Perceived Socioeconomic Status by Family Affluence Scale (FAS) |  |
| - High | 585 (62.2) |
| - Average | 305 (32.4) |
| - Low | 50 (5.3) |
| Educational institution type |  |
| - Public high schools | 639 (68.0) |
| - Private high schools | 301 (32.0) |

*Note.* *n=* frequency, *M*= mean, *SD=* standard deviation.

**Supplementary Table 2**

*Group comparisons of sociodemographic variables by gender (N= 940)*

| Variable | Male (n = 536) | Female (n = 404) | Test | p-value | Effect size |
| --- | --- | --- | --- | --- | --- |
| Age (years) | 15.50 (0.80) | 15.37 (0.72) | t-test | <.001 | Cohen’s d= 0.163 |
| Nationality |  |  |  |  |  |
| - Spanish, n (%) | 466 (86.9) | 357 (88.4) | χ² | .777 | Cramer’s V= 0.052 |
| - American (Central and South), n (%) | 40 (7.5) | 27 (6.6) |  |  |  |
| - Other European countries, n (%) | 11 (2.1) | 10 (2.5) |  |  |  |
| - Africa, n (%) | 12 (2.2) | 8 (2.0) |  |  |  |
| - Asia, n (%) | 7 (1.3) | 2 (0.5) |  |  |  |
| Perceived Socioeconomic Status by Family Affluence Scale (FAS) |  |  |  |  |  |
| - High, n (%) | 335 (62.5) | 250 (61.9) | χ² | .857 | Cramer’s V= 0.018 |
| - Average, n (%) | 171 (31.9) | 134 (33.2) |  |  |  |
| - Low, n (%) | 30 (5.6) | 20 (5.0) |  |  |  |
| Educational institute type |  |  | χ² |  |  |
| - Public high schools, n (%) | 370 (69.0) | 269 (66.6) |  | .059 | Φ (Phi coefficient) = 0.078 |
| - Private high schools, n (%) | 166 (31.0) | 135 (33.4) |  |  |  |

*Note.* Values are presented as n (%) for categorical variables and M (SD) for continuous variables. Effect sizes are indicated as Cohen’s d for continuous variables, Cramer’s V for categorical variables with more than two levels, and Φ (phi coefficient) for 2x2 categorical variables. χ² = chi-square test. *T-test* = independent samples t-test.

**Supplementary Table 3**

*Centrality measures per variable*

|  | | | | |
| --- | --- | --- | --- | --- |
|  | Network | | | |
| Variable | Betweenness | Closeness | Strength | Expected influence |
| BW | -0.699 | 0.278 | 0.325 | 1.099 |
| DS | 2.262 | 1.294 | 0.691 | -0.851 |
| De | -0.699 | -0.057 | -0.153 | 0.499 |
| E | -0.329 | 0.689 | 0.559 | 1.393 |
| IN | 0.596 | -1.481 | -0.512 | -0.250 |
| LC | 0.596 | 0.543 | 1.525 | -0.508 |
| PE | -0.329 | 0.744 | 0.315 | 0.683 |
| PP | -0.699 | -0.412 | -1.927 | -1.725 |
| WM | -0.699 | -1.599 | -0.823 | -0.341 |

*Note.* E = Engagement; PE = Positive Emotions; DS = Desire Savoring; PP = Pleasure Preservation; BW = Binge-Watching; De = Dependency; LC = Loss of Control; IN = Inhibition; WM = Working Memory.

**Supplementary Figure 1**

*Expected Influence of Nodes in the Total Sample*

*Note.* Expected influence values are normalized to facilitate comparison across nodes. E = Engagement; PE = Positive Emotions; DS = Desire Savoring; PP = Pleasure Preservation; BW = Binge-Watching; De = Dependency; LC = Loss of Control; IN = Inhibition; WM = Working Memory.

**Supplementary Figure 2**

*Edge Weight Stability of BWESQ-TEXI Networks by total sample*

*Note.* Edge weight stability was assessed via bootstrapped resampling (1,000 iterations). The red line represents the sample values, while the black line and gray area represent the bootstrap mean and the 95% confidence intervals, respectively. E = Engagement, PE= Positive Emotions, DS= Desire Savoring, PP= Pleasure Preservation, BW= Binge- Watching, De= Dependency, LC= Loss of Control, IN= Inhibition, WM = Working Memory. Lines represent 95% confidence intervals for each edge, illustrating the reliability of connections. Edge thickness in the original network reflects partial correlation magnitude.

**Supplementary Figure 3**

*Node Centrality Indices by Gender*

*Note.* E = Engagement, PE = Positive Emotions; DS = Desire Savoring; PP = Pleasure Preservation; BW = Binge-Watching, De = Dependency; LC = Loss of Control; IN = Inhibition; WM = Working Memory. Centrality estimates are displayed separately by gender, with red lines corresponding to men and blue lines corresponding to women.

**Supplementary Figure 4**

*Edge Weight Stability of BWESQ-TEXI Networks by gender*

*Note.* Upper panel represents men; right panel represents women. Edge weight stability was assessed via bootstrapped resampling. Lower edges represent negative partial correlations. E = Engagement, PE= Positive Emotions, DS= Desire Savoring, PP= Pleasure Preservation, BW= Binge Watching, De= Dependency, LC= Loss of Control, IN= Inhibition, WM = Working Memory. Lines represent 95% confidence intervals for each edge, illustrating the reliability of connections. Edge thickness in the original network reflects partial correlation magnitude.

**Supplementary Figure 5**

*Bootstrapped Stability of Node Centrality by Gender*

*Note.* The left panel shows bootstrapped difference tests for the male network, and the right panel shows the female network. Black boxes indicate statistically significant differences between node centrality measures (Strength, Betweenness, Closeness; p < .05), while grey boxes indicate non-significant differences. White diagonal values display the raw centrality stability coefficients. These plots confirm the robustness and internal consistency of the centrality rankings in both gender-specific networks. BWESQ’s dimension: E = Engagement, PE = Positive Emotions, PP = Pleasure Preservation, DS = Desire Savoring, BW = Binge-Watching, De = Dependency and LC = Loss of Control. TEXI’s dimension: IN = Inhibition and WM = Working Memory.

**Supplementary Figure 6.**

*Correlation Stability (CS) plot for males BWESQ-TEXI Network.*

*Note.* Correlation Stability (CS) plot depicting the robustness of centrality indices (Strength / Expected Influence) for binge-watching (BWESQ) and executive functioning (TEXI) dimensions in men. The X-axis represents the proportion of cases removed from the sample, and the Y-axis shows the correlation of centrality indices in the reduced sample with the original full sample. The shaded area illustrates the variability of centrality estimates across bootstrap resamples, reflecting how stable the estimates remain as participants are dropped. Values above 0.50 indicate acceptable stability, with higher values reflecting greater robustness. In this study, edge weights were highly stable (CS = 0.75), and strength centrality was acceptable (CS = 0.51).

**Supplementary Figure 7.**

*Correlation Stability (CS) plot for females BWESQ-TEXI Network.*

*Note.* Correlation Stability (CS) plot depicting the robustness of centrality indices (Strength / Expected Influence) for binge-watching (BWESQ) and executive functioning (TEXI) dimensions in women. The X-axis represents the proportion of cases removed from the sample, and the Y-axis shows the correlation of centrality indices in the reduced sample with the original full sample. The shaded area illustrates the variability of centrality estimates across bootstrap resamples, reflecting how stable the estimates remain as participants are dropped. Values above 0.50 indicate acceptable stability, with higher values reflecting greater robustness. In this study, edge weights were highly stable (CS = 0.75), and strength centrality was acceptable (CS = 0.51).

**Supplementary Figure 8.**

*Bridge Expected Influence in the BWESQ-TEXI Network for males.*

*Note.* Bridge Expected Influence (Bridge EI) scaled from 0 to 1, representing the relative importance of each node in connecting communities (clusters of nodes identified via EGA) within the BWESQ-TEXI network. Higher values indicate stronger bridging capacity. E = Engagement; PE = Positive Emotions; DS = Desire Savoring; PP = Pleasure Preservation; BW = Binge Watching; De = Dependency; LC = Loss of Control; IN = Inhibition; WM = Working Memory. Edge thickness reflects the magnitude of partial correlations, with red edges indicating negative associations.

**Supplementary Figure 9.**

*Bridge Expected Influence in the BWESQ-TEXI Network for females.*

*Note.* Bridge Expected Influence (Bridge EI) scaled from 0 to 1, representing the relative importance of each node in connecting communities (clusters of nodes identified via EGA) within the BWESQ-TEXI network. Higher values indicate stronger bridging capacity. E = Engagement; PE = Positive Emotions; DS = Desire Savoring; PP = Pleasure Preservation; BW = Binge Watching; De = Dependency; LC = Loss of Control; IN = Inhibition; WM = Working Memory. Edge thickness reflects the magnitude of partial correlations, with red edges indicating negative associations.
